# Evaluation of Solanaceous Species as Nonhost Trap Crops for *Globodera pallida*

**DOI:** 10.2478/jofnem-2023-0036

**Published:** 2023-09-01

**Authors:** Paige Hickman, Louise-Marie Dandurand

**Affiliations:** Department of Entomology, Plant Pathology, and Nematology, University of Idaho, Moscow, ID 83844

**Keywords:** *Globodera pallida*, hatching, management, pale cyst nematode, potato cyst nematode, Solanaceae, trap crop

## Abstract

*Globodera pallida*, the pale cyst nematode (PCN), is a quarantine pest of potato posing a serious threat to the Idaho potato industry. *Globodera pallida* only hatches in the presence of a hatching stimulus produced by a host plant or closely related species. In the absence of this hatching stimulus, *G. pallida* can remain viable in the soil for decades. A trap crop stimulates hatch of *G. pallida* but is a nonhost, which means the nematode cannot develop or reproduce. This study evaluated the trap crop potential of several solanaceous species by determining *G. pallida* host status and hatching effect of each species. The species under investigation included *Solanum aethiopicum*, *S. macrocarpon*, *S. quitoense*, *S. retroflexum*, and *S. douglasii*. All species were determined to be nonhosts of *G. pallida*. The most promising trap crop candidates with a hatching stimulatory effect comparable to potato were *S. quitoense* and *S. retroflexum.* Further research is needed to assess whether these species could be effective *G. pallida* trap crops under Idaho field conditions.

*Globodera pallida* (Stone) Behrens, the pale cyst nematode, is a regulated pest of potato in the United States. It originated in the Andes region but has since become widespread in many parts of the world (Grenier et al., 2010). *Globodera pallida* was first reported in Idaho in 2006 and was confirmed with morphological and molecular identification (Skantar et al., 2007). USDA-APHIS works to contain and eradicate *G. pallida* from infested fields in Idaho. Regulated acreage is not allowed back into potato production until it has undergone the extensive APHIS deregulation process. As of 2022, APHIS reports that 2,658 ha are regulated, of which 1,433 ha are considered infested (USDA-APHIS, 2022). *Globodera pallida* presents a major threat to the Idaho potato industry with potential for 80% yield loss in severe infestations (Contina et al., 2019), therefore eradication efforts are crucial. U.S. potato production was valued at $4 billion in 2021 with the United States ranking as the world's fifth top potato producer (US-NASS, 2022a; FAOSTAT, 2022). Idaho is the country's top potato-producing state (US-NASS, 2022b). In 2016, *G. pallida* was estimated to cause a net loss of $25.56 million from the Idaho industry (Koirala et al., 2020). Potatoes are the highest-valued crop in Idaho and thus, losses from infested acreage out of production cannot be completely offset by producing other crops (Koirala et al., 2020).

Several challenges are associated with control of *G. pallida*. Dormant second-stage juveniles (J2s) within eggs can survive inside cysts in the soil in the absence of a host for up to three decades (Turner, 1996). In order to hatch, the juveniles require a signal or hatching factor, which is typically only found in root exudates of its host plant, potato, and some closely related species (Masler and Perry, 2018). Fumigants and nematicides are the primary methods of control for *G. pallida*. Since the methyl bromide ban in 2015, current fumigation efforts rely on 1,3-dichloropropene (USDA-APHIS, 2022). Aside from fumigants, trap crops can be a valuable control strategy for *G. pallida*. Trap crops take advantage of hatching factors produced by nonhosts which then prevent development and reproduction of the nematode. Once hatched, the second stage juveniles (J2s) are highly vulnerable, and without being able to feed, they will die within days (Storey, 1984). A hatching factor is important for *G. pallida* control because decline in egg densities in absence of this hatching stimulus is low (Evans, 1993). *Globodera pallida* hatching factors are restricted to the Solanaceae family, which make solanaceous species of particular interest as trap crops (Ngala et al., 2021; Scholte, 2000).

*Solanum sisymbriifolium* Lam. is commonly known as litchi tomato or sticky nightshade. It originated in South America and is grown in small-scale production for its edible fruit (Moehninsi et al., 2015). *Solanum sisymbriifolium* is a well-studied successful trap crop of *G. pallida* because it is resistant and stimulates hatch, greatly reducing *G. pallida* densities (Mhatre et al., 2021; Scholte and Vos, 2005; Scholte, 2000). *Globodera pallida* reproduction on susceptible potato is reduced by 99% following *S. sisymbriifolium* (Dandurand and Knudsen, 2016). However, this species is considered a noxious weed in some regions of the world and is a known host of potato spindle tuber viroid (Fowkes et al., 2021). Numerous other solanaceous species have been screened as potential trap crops, but many do not offer complete resistance to *G. pallida* and thus do not meet requirements for *G. pallida* trap crops in Idaho (Chitambo et al., 2019; Scholte, 2000). *Solanum villosum* and *S. scabrum*, for example, cause high rates of *G. pallida* hatch but still allow some reproduction (Chitambo et al., 2019).

This study investigated several potential solanaceous species as trap crops for the Idaho population of *G. pallida*. Species of interest include *Solanum aethiopicum* L., *Solanum macrocarpon* L., *Solanum quitoense* Lam., *Solanum retroflexum* Dunal, and *Solanum douglasii* Dunal. *Solanum aethiopicum* and *S. macrocarpon* are closely related eggplant species that are cultivated crops in Africa (Han et al., 2021; Plazas et al., 2014). They have high nutrient content and both fruits and foliage can be consumed (Han et al. 2021). *Solanum aethiopicum* and *S. macrocarpon* are tropical species that typically grow best in well-drained soil and cannot tolerate cold (Han et al., 2021). Both of these species have been found to cause hatch of *G. pallida* in vitro (Ngala et al., 2021; Scholte, 2000).

*Solanum quitoense* is commonly known as naranjilla and is a perennial shrub producing edible fruits native to South America (Dennis et al., 1984). Naranjilla is grown and consumed primarily in Central America and some parts of South America such as Ecuador and Colombia; it is native to the Andes region (Acosta et al., 2009). The fruit is consumed fresh or is processed into juice or jam (Acosta et al., 2009). It is a subtropical plant which is not tolerant of high tropical temperatures nor temperate climates, and it is a perennial but usually cultivated as an annual due to issues with root-knot nematode, *Meloidogyne spp.* (Heiser and Anderson, 1999). There is evidence that *S. quitoense* is a nonhost that causes hatch of *G. pallida* (Scholte, 2000). *Solanum retroflexum* is an annual plant that is native to Africa and has been introduced to parts of the United States as a garden variety (Schilling, 1981). Commonly called wonderberry, it produces edible fruits. *Solanum retroflexum* grows best in well-drained soil and is not cold tolerant. It has not yet been evaluated as a trap crop for *G. pallida*.

*Solanum douglasii*, or greenspot nightshade, is native to Mexico and parts of the Southwestern U.S. and does not produce an edible crop (USDA-PLANTS, n.d.). It is present in different areas of the U.S. and its host status for *G. pallida* has been unknown. Other species of nightshade which are present in Idaho, including *Solanum physalifolium*, *S. nigrum*, and *S. triflorum*, were determined to be hosts of *G. pallida* (Boydston et al., 2010). Ultimately, the goal of this study was to assess the host status and hatching effect of some of these solanaceous species to determine whether they have potential as trap crops for control of *G. pallida* in Idaho.

## Materials and Methods

### Nematode Culture

*Globodera pallida* was originally obtained from infested fields in Shelley, Idaho. The identity was confirmed by morphological and molecular methods (Skantar et al., 2007). The nematode was reared on the susceptible potato ‘Desiree’ or ‘Russet Burbank’ under greenhouse conditions at 18°C and 16-hour light:8-hour dark photoperiod for 12 weeks. Dried cysts were recovered from soil by elutriator extraction, dried, and stored at 4°C until experimental use (USDA-APHIS, 2009).

### Plant Propagation

Seeds of the solanaceous species under investigation were acquired from the U.S. National Plant Germplasm System (NPGS) via GRIN-GLOBAL. The following seeds were received from NPGS in Fall 2019: *Solanum aethiopicum* (accession Grif 14165), *Solanum macrocarpon* (accession PI 441914), *Solanum quitoense* (accession PI 489701), *Solanum retroflexum* (accession PI 634755), and *Solanum douglasii* (accession W6 56881). Seeds were planted into potting mix and grown for four weeks following emergence. Susceptible potato positive controls included ‘Desiree’ and ‘Russet Burbank’, which were grown for four weeks as tissue cultured plantlets in standard media (Murashige and Skoog, 1962).

### Growing Conditions

Experiments were initiated when four-week-old tissue culture potato plantlets or seedlings from potting soil were transplanted into terracotta clay pots containing soil (Dandurand and Knudsen, 2016). Soil consisted of a 2:1 ratio of Lane Mountain 20/30 industrial silica sand to Prosser-series silt loam soil (WSU-IAREC, Prosser, WA) that had been dried and sieved through a 5 mm mesh (Dandurand and Knudsen, 2016). Soil was autoclaved twice for 90 min at 121°C prior to use. Greenhouse conditions were maintained between 16°C and 20°C with 60% relative humidity and a 16-hour light:8-hour dark photoperiod. Pots were watered daily to maintain soil moisture. Osmocote slow-release fertilizer (The Scotts Company, Marysville, OH) was applied at planting. Jack's Classic All Purpose 20-20-20 water soluble fertilizer (JR Peters Inc., Allentown, PA) was applied weekly. Bioworks SuffOil-X horticultural oil (Bioworks, Victor, NY) was applied weekly to prevent thrips infestation.

### Host Assay

The host assay was performed in 10-cm-diameter terra cotta pots containing approximately 500 g soil. Pots were inoculated with the Idaho population of *Globodera pallida* that had been reared on susceptible potato under greenhouse conditions, and had completed diapause. Cysts were sealed within sterile 6 cm^2^ nylon mesh (McMaster Carr, Elmhurst, IL) bags with 250 μm opening, with edges sealed by a hand sealer (ULINE Tabletop Impulse Sealer). Prior to inoculation, cysts were surface sterilized in 0.3% hypochlorous bleach for 5 min followed by five thorough rinses with sterile deionized water (Nour et al., 2003). The initial infestation rate was approximately 5 eggs/g soil, or 12 cysts per bag with an average of 200 eggs/cyst. Cyst bags were placed in the root zone of transplanted seedlings. Susceptible potatoes ‘Russet Burbank’ and ‘Desiree’ were included as positive controls. Bare soil unplanted pots were included as negative controls. Six replicate pots were grown in randomized complete block design for 12 weeks. The experiment was then terminated. Plants were cut off at the soil surface. Soil and root samples were dried for two weeks and then collected for cyst extraction. The host assay trial was repeated.

Cysts were extracted using the elutriator system (USDA-APHIS, 2009). Following extraction, cysts were floated in acetone to further remove debris (Brodie et al., 1976). Samples were then examined with a dissecting scope (Leica M80, Leica Microsystems, Wetzlar, Germany) where any progeny cysts were counted. A species that had zero progeny cysts in all replicates was determined to be a nonhost of *G. pallida.* Cyst bags were recovered from the host assay and three cysts per replicate were crushed in 100 μl sterile deionized water in a well of a 96-well plate (VWR® tissue culture plates, Radnor, PA) to determine average remaining encysted eggs. Eggs were counted using an inverted microscope (Leica DMi1, Leica Microsystems, Wetzlar, Germany). Remaining encysted egg viability was determined by the acridine orange staining method (Pillai and Dandurand, 2019). Non-viable eggs were counted with an inverted fluorescent microscope (Leica DMi8, Leica Microsystems, Wetzlar, Germany). Remaining viable eggs were calculated as total eggs – stained nonviable eggs. Percentage viability was calculated as (total eggs – stained nonviable eggs) / total eggs × 100.

### Root Exudates Collection

Hatching assays were conducted using root exudates. Plants for root exudate collection were grown in 15-cm-diameter terra cotta pots containing 1,200 g soil. Susceptible potatoes ‘Desiree’ and ‘Russet Burbank’ were included as positive controls. An unplanted bare soil pot was included as a negative control. Four replicate pots were grown in randomized complete block design. Root exudates were collected at four weeks and at six weeks, following transplant into soil. Pots were not watered 24 hours prior to root exudate collection. Root exudates were collected by soil leaching, modified from Widdowson and Wiltshire (1958). Soil was first saturated with deionized water one hour prior to diffusate collection. Pots were then balanced on plastic cups placed beneath their drainage holes. Deionized water was slowly added to each pot until approximately 30 ml of diffusate was collected in the cup. The diffusate samples were then vacuum filtered, first through a 0.45-μm-pore filter (Corning disposable vacuum filter, Corning, NY) to remove soil particles and debris and then through a 0.22-μm-pore filter to remove microbes (Corning disposable vacuum filter, Corning, NY). The resulting root exudates were then frozen at −20°C until use.

### Hatching Assay

In vitro hatching assays were performed within two months of collecting the exudates. The assays utilized cysts of the Idaho *G. pallida* population as described above. Cysts were surface sterilized in 0.3% hypochlorous bleach for five min before being thoroughly rinsed five times in sterile deionized water (Nour et al., 2003). Cysts were then hydrated for 48 hours in a 1:1 solution of sterile deionized water and 0.5% gentamicin. Cysts were then crushed to release the eggs. Approximately 100 eggs in 100 μl of 0.5% gentamicin solution were pipetted into wells of a 96-well plate. Initial eggs containing juveniles and initial released juveniles were counted using an inverted microscope (Leica DMi1, Leica Microsystems, Wetzlar, Germany). Root exudate, 100 μl, was applied to each well. There were three wells (technical replicates) per replicate of diffusate. Plates were incubated at 18°C for two weeks. Juveniles were then counted. The percentage hatch was calculated using the following equation: (Final J2s-Initial J2s) / Initial Eggs × 100. The root exudate collection and hatching assay experiment was repeated.

### Data Analysis

Statistical analysis was performed using the SAS statistical package (SAS Institute Inc., Cary, NC). Hatch, viability, and egg densities were analyzed with a generalized mixed linear model (PROC GLIMMIX) and means separation by least squares means. To meet the assumptions of a normal distribution, the host assay progeny cyst data was transformed as log_10_ (x +1) in order to be analyzed using PROC GLM and means separation by Tukey's HSD.

## Results

### *Globodera pallida* Host Assay

Reproduction of *G. pallida* was high in the susceptible potato varieties ‘Desiree’ and ‘Russet Burbank’ but no reproduction was observed in any of the solanaceous species tested (*P* < 0.0001) (Table 1). Trial two susceptible potato varieties also had significant cyst reproduction (Table 2). Like the bare soil negative control, all solanaceous species investigated, other than potato, had zero cysts in all replicates in both trials and are thus considered nonhosts for *G. pallida*.

**Table 1.** Trial one *Globodera pallida* progeny cysts, remaining encysted eggs recovered from cyst bags, viability of remaining encysted eggs, viable remaining encysted eggs, and determined host status for *G. pallida.* Data presented are means of six replicates. Significant differences are denoted by different letters in the columns based on least squares means at α = 0.05.

**Species**	**Progeny Cysts/Pot**	**Remaining Encysted Eggs/Cyst**	**Viability of Remaining Eggs/Cyst**	**Viable Remaining Encysted Eggs/Cyst**	**Host Status for *Globodera pallida***
*Solanum aethiopicum*	0 c	160.4 a	65.2% abc	81.9 a	Nonhost
*Solanum douglasii*	0 c	135.8 a	62.4% abc	58.8 b	Nonhost
*Solanum macrocarpon*	0 c	147.8 a	52.6% bc	46.7 bc	Nonhost
*Solanum quitoense*	0 c	96.9 b	48.0% c	32.8 c	Nonhost
*Solanum retroflexum*	0 c	91.2 b	61.5% abc	44.9 bc	Nonhost
Bare Soil	0 c	158.4 a	73.7% a	89.1 a	Nonhost
*Solanum tuberosum* ‘Desiree’	369.7 a	77.9 bc	66.1% ab	43.9 bc	Host
*Solanum tuberosum* ‘Russet Burbank’	265.5 a	70.9 c	61.3% abc	33.8 c	Host

**Table 2.** Trial two *Globodera pallida* progeny cysts, remaining encysted eggs recovered from cyst bags, viability of remaining encysted eggs, viable remaining encysted eggs, and determined host status for *G. pallida*. Data presented are means of six replicates. Significant differences are denoted by different letters in the columns based on least squares means at α = 0.05.

**Species**	**Progeny Cysts**	**Remaining Encysted Eggs/Cyst**	**Viability of Remaining Eggs**	**Viable Remaining Encysted Eggs**	**Host Status for *Globodera pallida***
*Solanum aethiopicum*	0 b	147.5 a	61.2%	62.8 b	Nonhost
*Solanum douglasii*	0 b	133.7 ab	70.0%	51.2 bcd	Nonhost
*Solanum macrocarpon*	0 b	133.2 ab	55.3%	57.1 bc	Nonhost
*Solanum quitoense*	0 b	108.9 b	59.9%	63.7 b	Nonhost
*Solanum retroflexum*	0 b	77.7 c	65.5%	43.5 d	Nonhost
Bare Soil	0 b	148.4 a	65.7%	93.7 a	Nonhost
*Solanum tuberosum* ‘Desiree’	57.2 a	81.1 c	63.7%	44.4 cd	Host
*Solanum tuberosum* ‘Russet Burbank’	64.8 a	80.3 c	64.9%	43.5 d	Host

Remaining encysted eggs recovered from cysts used in inoculation in the host assay revealed that certain solanaceous species caused significantly more hatch than others (*P* < 0.0001) (Table 1). The bare soil control (158.4 ± 9.9 remaining eggs per cyst) was statistically the same as *S. aethiopicum* (160.4 ± 9.6 remaining eggs per cyst), *S. douglasii* (135.8 ± 8.1 remaining eggs per cyst), and *S. macrocarpon* (147.8 ± 8.2 remaining eggs per cyst). The susceptible potato varieties ‘Desiree’ (77.9 ± 4.7 remaining eggs per cyst) and ‘Russet Burbank’ (70.9 ± 12.7 remaining eggs per cyst) had about 50% less remaining encysted eggs than the bare soil, and were statistically the same as *S. quitoense* (96.9 ± 7.9 remaining eggs per cyst) and *S. retroflexum* (91.2 ± 9.3 remaining eggs per cyst). This indicates that *S. quitoense* and *S. retroflexum* induced hatch and thus may be useful as trap crops while *S. aethiopicum, S. douglasii,* and *S. macrocarpon* did not induce hatch and are not suitable trap crops. Data from trials one and two could not be combined due to interactions between trial and treatment. The trial two host assay had the same trends except that in this trial, the numbers of remaining encysted eggs with *S. quitoense* were significantly greater than the susceptible potato controls by 26% (Table 2).

In trial one, the percentage viability of the remaining eggs per cyst significantly differed in some treatments (*P* = 0.0014) (Table 1). Percentage viability of remaining eggs for the bare soil treatment (73.7% ± 1.7) was not statistically different for *S. aethiopicum* (65.2% ± 2.2), *S. douglasii* (62.4% ± 5.7), *S. retroflexum* (61.5% ± 2.9), and susceptible potato controls ‘Desiree’ (66.1% ± 5.0) and ‘Russet Burbank’ (56.3% ± 5.7). The percentage viability of remaining eggs for *S. quitoense* (48.0% ± 2.3) was significantly less than that of the bare soil control and susceptible potato ‘Desiree’ by 22%. The percentage viability of remaining eggs for *S. macrocarpon* (52.6% ± 5.3) was significantly less than that of the bare soil control. Compared to the controls, *S. macrocarpon* and *S. quitoense* are the only species which appear to affect egg viability. Data from trials one and two could not be combined due to interactions between trial and treatment. In trial two, viability of remaining encysted eggs was not significantly different among treatments (Table 2).

The mean remaining viable eggs per cyst was also significantly different in some treatments (*P* < 0.0001) (Table 1). The greatest remaining viable eggs were in the bare soil control (89.1 ± 5.0 viable eggs per cyst) and *S. aethiopicum* (81.9 ± 6.0 viable eggs per cyst). The greatest reduction in viable eggs was in the susceptible potato controls ‘Russet Burbank’ (33.8 ± 3.7 viable eggs per cyst) and ‘Desiree’ (43.9 ± 5.3 viable eggs per cyst), *S. macrocarpon* (46.7 ±10.1 viable eggs per cyst) and *S. quitoense* (32.8 ± 4.2 viable eggs per cyst). Compared to bare soil, *S. macrocarpon* and *S. quitoense* reduced viable eggs by 48% and 63%, respectively. *Solanum retroflexum* and *S. douglasii* were statistically the same as that of *S. macrocarpon* and ‘Desiree’. The host assay in trial two was similar to trial one except that viable eggs were less in the bare soil control than with *S. aethiopicum* (Table 2). Also, *S. quitoense* did not reduce viable eggs as much as the potato (Table 2).

### Hatching Assay

**Figure 1:**
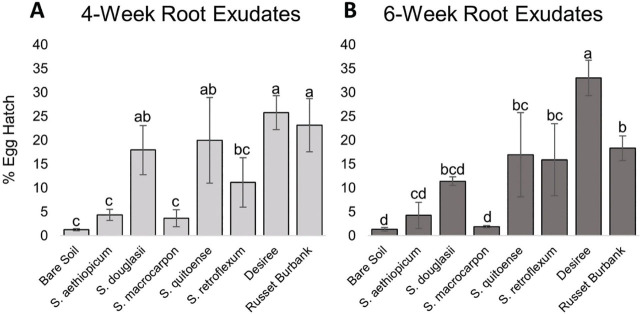
Trial one mean percentage egg hatch two weeks after root exudate application. A) Percentage egg hatch for root exudates collected at four weeks of growth. B) Percentage egg hatch for root exudates collected at six weeks of growth. Standard error of the means is indicated by the bars. Different letters indicate significantly different means based on least squares means at α = 0.05.

The in vitro hatching assay using root exudates collected at four weeks revealed significant differences in hatch among the treatments (*P* = 0.0021) (Fig. 1A). *Solanum quitoense* caused 20% egg hatch and *S. douglasii* caused 18% egg hatch and were statistically the same as the susceptible potato controls ‘Desiree’ with 26% hatch and ‘Russet Burbank’ with 23% hatch. The bare soil control diffusate had approximately 1% egg hatch. *Solanum aethiopicum* with 4% hatch, *S. macrocarpon* with 4% hatch, *S. retroflexum* with 11% hatch was not significantly different than the bare soil. The *S. retroflexum* hatch was also not significantly different than the *S. quitoense* and *S. douglasii* hatches.

The hatching assay using root exudates collected at six weeks also showed significant differences among treatments (*P* = 0.0013) (Fig. 1B). The susceptible potato control ‘Desiree’ caused 33% hatch while ‘Russet Burbank’ caused 18% hatch. *Solanum quitoense* with 17% hatch, *S. retroflexum* with 16% hatch, and *S. douglasii* with 11% hatch caused hatch statistically the same as that of ‘Russet Burbank’. The bare soil control hatch rate was 1%. *Solanum aethiopicum* with 4% hatch, *S. macrocarpon* with 2% hatch, and *S. douglasii* with 11% hatch were not significantly different than the bare soil control. *Solanum douglasii* hatch was also not significantly different from ‘Russet Burbank’.

**Figure 2:**
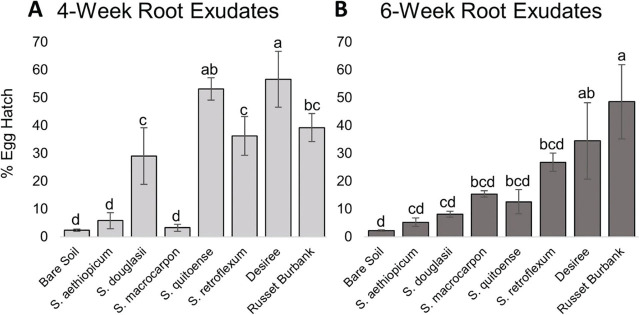
Trial two mean percentage egg hatch two weeks after root exudate application. A) Percentage egg hatch for root exudates collected at four weeks of growth. B) Percentage egg hatch for root exudates collected at six weeks of growth. Standard error of the means is indicated by the bars. Different letters indicate significantly different means based on least squares means at α = 0.05.

Trials one and two of the hatching assays could not be combined due to interactions between treatment and trial. The trial two hatching assay showed similar trends to trial one for the root exudates collected at 4 weeks, in that *S. douglasii* (29% hatch), *S. quitoense* (53% hatch), and *S. retroflexum* (36% hatch) caused hatch like that of the susceptible potato controls ‘Desiree’ (57% hatch) and ‘Russet Burbank’ (39% hatch) (Fig. 2). However, in trial 2 for the root exudates collected at 6 weeks, *S. douglasii* (8% hatch) was not significantly different from the bare soil (2% hatch) (Fig. 2).

## Discussion

This study confirms the findings of previous studies that *S. quitoense*, *S. macrocarpon*, and *S. aethiopicum* are nonhosts for *G. pallida* (Scholte, 2000). It was also determined for the first time that *S. retroflexum* and *S. douglasii* are nonhosts for *G. pallida*.

The remaining encysted eggs from the cysts originally used to inoculate the host assay were the highest in the bare soil control, as there was no hatching stimulus for *G. pallida* in bare soil. Based on remaining encysted eggs, *S. quitoense* and *S. retroflexum* caused in vivo hatch comparable to that of potato. *Solanum douglasii*, *S. macrocarpon*, and *S. aethiopicum* were not effective in reducing eggs and were comparable to the bare soil treatment. In the in vitro hatch assays, *S. quitoense*, *S. retroflexum*, and *S. douglasii* consistently caused hatch comparable to that of potato. In contrast, *S. macrocarpon* and *S. aethiopicum* did not induce hatch more than the bare soil treatment. There is some discrepancy between the percentage hatch caused by the root exudates in vitro and the average encysted eggs remaining from the host assays. For example, *S. douglasii* induced more hatch in the in vitro hatch assay than during the host assay. However, induction of hatch while directly being exposed to the plant may be impacted by plant growth, duration of exposure, and a variety of other factors (Ngala et al., 2021). The host assay may be a more accurate in vivo representation of what would happen to PCN if these plants were to be planted in an infested field.

Ultimately, our results indicate that, of the species investigated, *S. retroflexum* and *S. quitoense* show potential as *G. pallida* trap crops by causing hatch comparable to potato. *Globodera pallida* hatching response to root exudates can also be affected by plant age (Byrne et al., 2001). In a study comparing hatching of *G. rostochiensis* and *G. pallida*, Byrne et al. (2001) determined that *G. pallida* hatch was greater with exudates collected from plants that were less that than 38 days old while *G. rostochiensis* hatch was higher when exposed to exudates from older plants. This may account for why hatch varied in the root exudates collected at four weeks compared to the exudates collected at six weeks. To increase hatch of *G. pallida* and optimize the impact of the trap crop, growth of the trap crop throughout the growing season may be essential, especially because *G. pallida* tends to hatch more gradually over time compared to *G. rostochiensis* (Whitehead, 1992).

The effect of potential trap crops on *G. pallida* densities would need to be evaluated in Idaho field conditions to determine their impact on *G. pallida* in infested fields. Although the species under investigation in this study are best adapted for warm tropical climates, they may be suited to the irrigated farmland practices found in the region of the infested fields. *Solanum aethiopicum* and *S. macrocarpon* are adapted for tropical climates of Africa (Han et al., 2021), while *S. retroflexum* is cultivated as a garden variety in the southern U.S. (Schilling, 1981). Both may be suited to the hot growing conditions of the Snake River Valley. However, *S. quitoense* is not tolerant of temperate climates (Heiser and Anderson, 1999). *Solanum douglasii* is also more adapted for climates of the southwestern U.S. and is considered to be a weed. Further studies on agronomic characteristics and requirements are needed to assess their suitability for use in Idaho. If these species are not currently adapted for Idaho's long day length and climate, future studies may look to plant breeders to create varieties better adapted for Idaho.

Future studies focusing on fractionating diffusate and determining the compounds inducing hatch may provide novel control strategies. Guerrieri et al. (2021) found hatch to be caused by solanoeclepin A, which is produced by several solanaceous species including *S. sisymbriifolium* (Guerrieri et al., 2021). The rate of hatching induced by some root exudates was found to be positively correlated with the concentration of solanoeclepin A (Guerrieri et al., 2021). Certain solanaceous species may induce greater hatch than others because their concentration of hatching stimulus is higher. Much research remains to be done regarding nonhost solanaceous trap crops of *G. pallida*. If the solanaceous species under investigation in this study are not suitable for Idaho conditions, then perhaps the hatching factors in their root exudates may still be of use for *G. pallida* eradication efforts in Idaho if they can be identified and developed for soil application. Furthermore, understanding the molecular basis of plant defenses from trap crops may aid in development of resistance. Nevertheless, these trap crops may be highly suitable for use in tropical areas that struggle to find appropriate solutions for *Globodera spp.* management.
